# The effect of body weight-supported overground gait training for patients with Parkinson’s disease: A retrospective case-control observational study

**DOI:** 10.1371/journal.pone.0254415

**Published:** 2021-07-20

**Authors:** Yasuki Koyanagi, Isato Fukushi, Masatoshi Nakamura, Kouji Suzuki, Nobuhito Oda, Takashi Aita, Hareaki Seki

**Affiliations:** 1 Department of Rehabilitation, National Hospital Organization Sendai Medical Center, Sendai, Japan; 2 Department of Neurology and Rehabilitation, National Hospital Organization Iwaki Hospital, Iwaki, Japan; 3 Faculty of Health Sciences, Uekusa Gakuen University, Chiba, Japan; 4 Clinical Research Center, Murayama Medical Center, Musashimurayama, Japan; 5 Institute for Human Movement and Medical Sciences, Niigata University of Health and Welfare, Niigata, Japan; Universidade Federal do Rio Grande do Sul, BRAZIL

## Abstract

**Objective:**

To evaluate the effects of body weight-supported overground gait training (BWSOGT) on motor abilities, such as gait and balance, in patients with Parkinson’s disease (PD).

**Design:**

Retrospective case-controlled observational study with a 4-week follow-up.

**Setting:**

Inpatient rehabilitation.

**Participants:**

We selected 37 of 68 patients with PD. Inclusion criteria were (1) Hoehn & Yahr stage II–IV, (2) no medication adjustment during the study period, (3) at least 1 week since last medication adjustment, and (4) ability to walk more than 10 meters on their own. Exclusion criteria were (1) cerebrovascular disease or other complications affecting movement, (2) difficulty in measurement, (3) early discharge, (4) medication change during the study, and (5) development of complications.

**Interventions:**

Patients were divided into two groups. Patients in Group I underwent 20 minutes of BWSOGT with a mobile hoist in addition to the standard exercises; Group II performed 20 minutes of gait training in place of BWSOGT. In both groups, training was performed for a total of 15 times/4 weeks.

**Main outcome measure(s):**

Participants were evaluated using the Unified Parkinson’s Disease Rating Scale total, part II, and part III; 10-m walk test; velocity; stride length; 6-minute walk test; timed up and go test; Berg Balance Scale; and freezing of gait before and after the intervention.

**Results:**

There were significant decreases in the Unified Parkinson’s Disease Rating Scale total, part II, and part III in both groups; however, 6-minute walk test, timed up and go test, and freezing of gait results only improved in Group I.

**Conclusions:**

BWSOGT for patients with PD improves gait ability and dynamic balance more than standard gait training.

## Introduction

Parkinson’s disease (PD) is a progressive disease characterized by degeneration and loss of neuromelanin in parts of the brainstem, including the substantia nigra pars compacta of the midbrain and locus coeruleus [1]. Patients with PD often present with motor symptoms such as bradykinesia, muscular rigidity, postural reflex disturbances, and tremor, as well as autonomic dysfunction, mental disorder, cognitive disorder, and sleep disorder [1].

Exercise therapy for patients with PD is effective in improving physical function [2, 3] and is recommended in the treatment of PD in combination with pharmacotherapy [4]. Although it has been reported that gait and balance disorders in patients with PD are difficult to treat [5, 6], it has recently been reported that gait practice using external cueing [7] and body weight-supported treadmill training (BWSTT) improves gait and balance ability in patients with PD [8–10].

It has been reported that gait training in patients with PD is more effective when performed in situations similar to those faced in daily life [11] and that treadmill gait training with audiovisual stimulation has a greater effect [12]. However, in normal treadmill gait training, unlike in situations faced in daily life, the patient does not experience a visual change of scenery while walking [13]. To compensate for the lack of visual change during BWSTT, we focused on body weight-supported overground gait training (BWSOGT). BWSOGT is a form gait training performed on the floor that is partially body weight-supported using a walking hoist, which enables the performance of gait training on a floor in a state closer to that in daily life situations than with training on a treadmill because of dynamic visual cues during gait training. However, there have been no reports on the effect of BWSOGT on motor ability in patients with PD.

The purpose of this case-control study was to elucidate the effects of BWSOGT on motor abilities such as gait and balance in patients with PD.

## Materials and methods

This study obtained the approval of the Ethics Committee of Iwaki Hospital and was conducted in accordance with the Declaration of Helsinki.

### Participants

**Fig 1:**
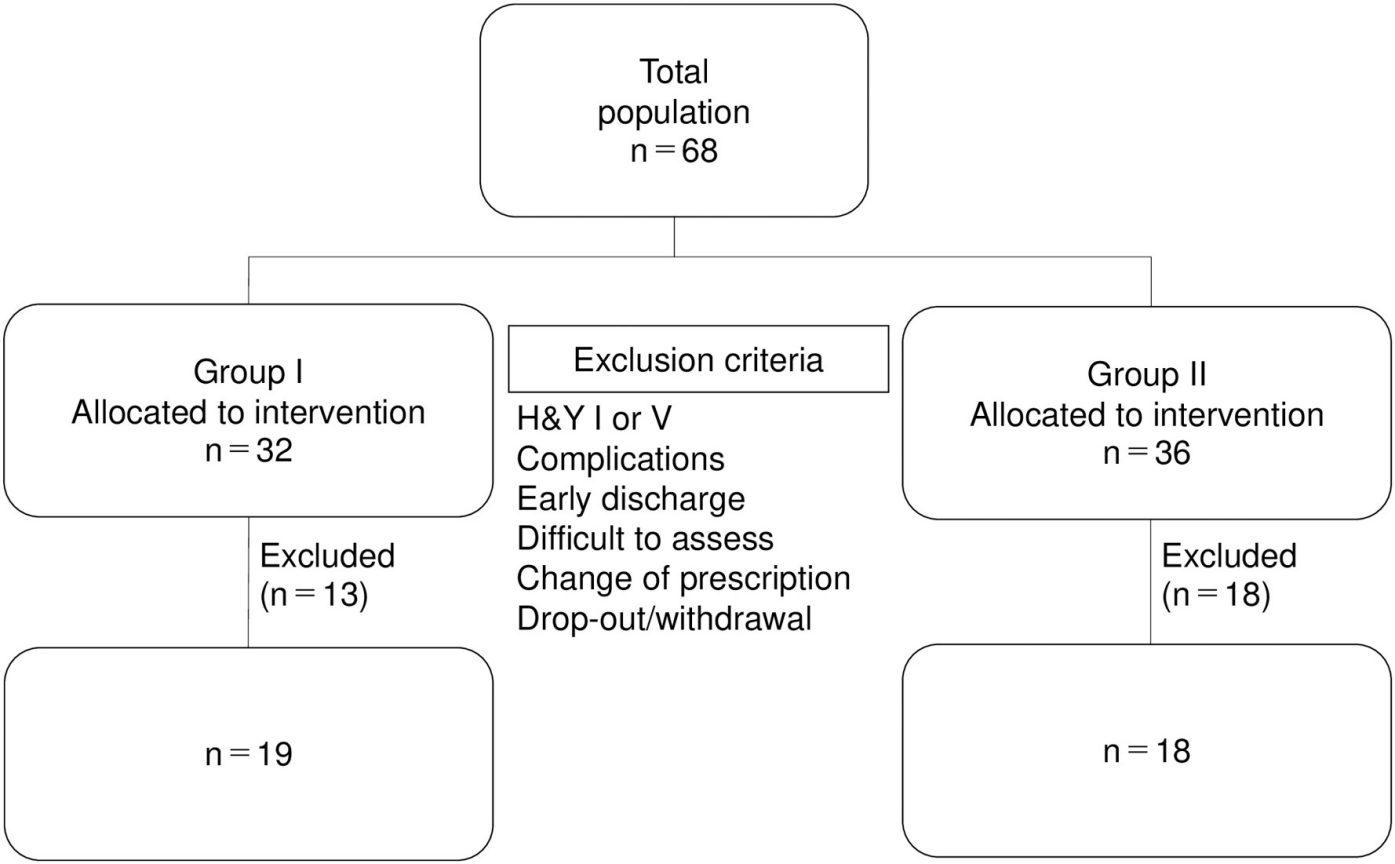
Flow chart showing participant selection with exclusion criteria.

This study included 68 patients with PD admitted to Iwaki Hospital from 1 April 2018 to 1 April 2019 who gave written informed consent to participate rehabilitation. After this study was approved by the ethics committee, we carried out this retrospective study between December 1, 2019 and March 31, 2020. This study used retrospective data obtained from our database and the requirement of written informed consent was waived by the ethics committee. Inclusion criteria were: (1) Hoehn & Yahr (H&Y) stage II–IV, (2) no medication adjustment during the study period, (3) at least 1 week after the last medication adjustment, and (4) ability to walk more than 10 meters on their own. Exclusion criteria were: (1) cerebrovascular disease or other complications affecting movement, (2) difficulty in measurement, (3) early discharge, (4) medication change during the study, and (5) development of complications. Thirty-seven patients with PD were selected. Patients admitted during the year when a mobile hoist was introduced performed BWSOGT at the discretion of the treating physician, and patients admitted during the year when a mobile hoist was not introduced performed gait training. The BWSOGT group was retrospectively divided into Group I and the gait training group into Group II, and the effects of the intervention were examined in 19 Group I patients (mean age 71.6 years, seven men) and 18 Group II patients (mean age 72.7 years, nine men) before and after the intervention (Fig 1). After calculating the sample size required for a split-plot analysis of variance (ANOVA) (effect size = 0.40 [large], α error = 0.05, and power = 0.80) using G* power 3.1 software (Heinrich Heine University, Düsseldorf, Germany) on the basis of a previous study [14], we determined that more than 14 participants were required for this study.

### Protocol

Patients in Group I were given 20 minutes of BWSOGT with a mobile hoist in addition to standard exercises, while Group II performed 20 minutes of gait training in place of the BWSOGT. Both groups underwent training for a total of 15 times/4 weeks. BWSOGT was performed using the Ropox Lifting Systems/All-in-One Walking Hoist (Ropox, Naestved, Denmark) with 20% of body weight supported, in accordance with previous studies [14, 15]. BWSOGT and gait training were performed on the flat floor of the training room, and the patients walking at the maximum possible speed were given verbal instructions on stride length. Additionally, BWSOGT was performed with a walking hoist towed from the front to elicit a faster gait in the patient. Standard exercises included stretching, muscle strengthening exercises, and balance exercises for 25 minutes, in accordance with previous studies [14, 16, 17]. These were the standard of care at our hospital, and patients who did not give consent to participate in this study received the same care.

### Outcome

The Unified Parkinson’s Disease Rating Scale (UPDRS) total, part II, part III, 10-m walk test (10MWT), velocity, stride length, 6-min walk test (6MWT), timed up and go test (TUG), Berg Balance Scale (BBS), and freezing of gait (FOG) were scored the day before the intervention and the day after the intervention was completed. All measurements were conducted during patients’ “on time.” Measurements were retrospectively collected from the electronic medical records of Iwaki Hospital.

The UPDRS is a standardized assessment criterion for PD that consists of four parts (Part I: Mental, Part II: Activity of Daily Living (ADL), Part III: Motor, and Part IV: Complications) for a total of 42 assessment items. We used the UPDRS total, ADL, and exercise items in this study.

The 10MWT was carried out on a 16-m flat walking path with 3 m of auxiliary path at the start and end; the walking time and number of steps were recorded. Measurements were made with patients walking at a comfortable velocity, and the average of two measurements was used. Walking velocity in m/s was calculated as the time taken divided by 10, and the stride length was the distance from the position beyond the start line to the position beyond the end line divided by the number of steps.

Results for the TUG were measured as the time taken to stand up from an armchair, walk to a marker 3 m away, change direction, and return to the chair. Measurements were made with patients walking at a comfortable velocity, and the average of two measurements was used [18].

Results for the 6MWT were measured as the greatest distance that could be covered walking over 6 min on a 30-m walking path in accordance with the guidelines of the American Thoracic Society [19–21].

The BBS is a comprehensive balance performance evaluation battery consisting of 14 items. Each item is rated on a scale of 0 to 4, with a maximum score of 56 [22].

The FOG was assessed using UPDRS 14 (Freezing When Walking) on a 5-point scale from 0 to 4, with higher values indicating more severe symptoms.

### Statistical analysis

IBM SPSS Statistics version 24.0 (IBM Corp, Armonk, NY, USA) was used to conduct statistical analyses. Between-group sex differences were assessed using chi-square tests. The normality of the distribution of characteristics and baseline values was confirmed using the Shapiro–Wilk test; the duration of PD, H&Y stages, Mini-Mental State Examination (MMSE), and levodopa dose were not normally distributed. Data that followed a normal distribution were analyzed using unpaired t-tests and shown as mean ± standard deviation (SD). Data that did not follow a normal distribution were analyzed using the Mann–Whitney U test and are shown as median (25–75%).

Moreover, the Shapiro–Wilk test confirmed that almost all data before and after the intervention were normally distributed. For all variables, a split-plot analysis of variance (ANOVA) using two factors (time [PRE vs. POST evaluation] and group [Group I vs. Group II]) was used to determine the presence of interactions and main effects. If there was a significant interaction effect, the change between PRE and POST was calculated to clarify the differences in the effect of BWSOGT, and these changes between Group I and Group II were compared using unpaired t-tests. Additionally, if there was no significant interaction effect, the main effect of time was investigated using a split-plot ANOVA. The effect size (ES) was calculated as the difference in the mean value between PRE and POST divided by the pooled SD in each group [23], with an ES of 0.00–0.19, 0.20–0.49, 0.50–0.79, and ≥0.80 being considered trivial, small, moderate, and large, respectively. Furthermore, we calculated the ES of the group difference in change between PRE and POST as the difference in the mean value between Group I and Group II divided by the pooled SD. Differences were considered statistically significant at an alpha level of p < 0.05. Descriptive data are shown as mean ± SD.

## Results

Of the 68 patients recruited to this study, 18 (5 in Group I and 13 in Group II) were excluded because of their H&Y staging (stage I) with preserved walking ability [24–26] or complications affecting exercise, such as cerebrovascular disease, and 13 (8 in Group I and 5 in Group II) were excluded because of early discharge, difficulty in evaluation, medication change, or complications occurring during the intervention. Finally, 37 patients (19 in Group I and 18 in Group II) were included in the study (Fig 1). All patients included in this study were able to perform the training sessions and PRE/POST measurements. No adverse events were reported during the program.

The characteristics of patients, MMSE score, levodopa, and UPDRS score at the PRE evaluation are shown in Table 1. There were no significant differences in characteristics, MMSE score, levodopa, or UPDRS score at PRE evaluation.

**Table 1:** Subjects’ clinical characteristics.

	Group I	Group II	P value
	*N* = 19	*N* = 18	
Age (year)	71.6±5.1	72.7±4.4	0.50
Height (m)	1.55±0.12	1.57±0.12	0.58
Weight (kg)	53.1±10.0	54.4±12.5	0.75
Sex (male)	7 (36.8%)	9 (50.%)	0.43
Duration of PD (year)	7 (5–13)	7.5 (4.25–10.5)	0.73
H&Y stages	3 (2–4)	3 (2–4)	1.00
MMSE score	26 (20–28)	24.5 (20–27.5)	0.68
UPDRS total score	55.9±15.0	46.7±16.3	0.09
UPDRS part II score	13.7±3.9	10.8±5.4	0.08
UPDRS part III score	33.9±11.5	27.8±8.9	0.09
Levodopa (mg)	400 (375–450)	400 (300–400)	0.22

NOTE. Values are mean ± SD, n (%) or median (25–75%)

Abbreviations: H&Y, Hoehn and Yahr; MMSE, Mini-Mental State Examination; UPDRS, Unified Parkinson’s Disease Rating Scale.

All variables in both Group I and Group II are shown in Table 2. The split-plot ANOVA indicated significant interaction effects for UPDRS total, part II, part III, TUG, 6MWT, and FOG score. Moreover, the significant interaction effects on walking velocity, stride length, and BBS score did not reveal significant differences (p = 0.06, p = 0.07, and p = 0.10, respectively); there were main effects of time factor for these variables (F = 13.2, p < 0.01; F = 9.5, p < 0.01; F = 24.9, p < 0.01, respectively).

**Table 2:** Functional outcome by type of rehabilitation.

	Group I		Group II		Interaction effect	
	PRE	POST	PRE	POST	F value	P Value
UPDRS total score	55.9±15.0	45.5±16.2	46.7±16.3	42.5±16.8	11.5	<0.01
	d =	0.67	d =	0.26	η_p_^2^ =	0.25
UPDRS part II score	13.7±3.9	10.8±4.5	10.8±5.4	9.6±5.3	8.0	<0.01
	d =	0.68	d =	0.23	η_p_^2^ =	0.19
UPDRS part III score	33.9±11.5	28.2±10.8	27.8±8.9	25.8±9.3	8.1	<0.01
	d =	0.52	d =	0.21	η_p_^2^ =	0.19
Velocity (m/s)	0.87±0.28	1.02±0.22	0.94±0.27	0.99±0.25	3.7	0.06
	d =	0.62	d =	0.18	η_p_^2^ =	0.10
Stride length (m)	0.85±0.25	0.97±0.19	0.93±0.27	0.96±0.23	3.6	0.07
	d =	0.55	d =	0.12	η_p_^2^ =	0.09
TUG (s)	22.0±14.0	17.1±10.9	16.1±7.2	15.4±5.7	4.2	<0.05
	d =	0.39	d =	0.11	η_p_^2^ =	0.11
BBS score	43.5±7.7	47.1±6.8	47.7±5.7	49.4±4.8	2.9	0.10
	d =	0.49	d =	0.33	η_p_^2^ =	0.08
6MWT (m)	272.1±114.0	325.5±107.7	305.7±86.0	319.8±107.0	4.3	<0.05
	d =	0.48	d =	0.15	η_p_^2^ =	0.11
FOG score	1.4±0.9	0.8±0.9	0.8±0.8	0.7±0.7	6.6	<0.05
	d =	0.6	d =	0.15	η_p_^2^ =	0.16

Abbreviations: UPDRS, Unified Parkinson’s Disease Rating Scale; TUG, timed up and go test; BBS, Berg Balance Scale; 6MWT, 6-min walking test; FOG, freezing of gait.

Regarding variables with a significant interaction effect, the changes before and after intervention in both Group I and Group II were calculated and are shown in Table 3. The unpaired t-test revealed that changes in Group I were significantly larger than those in Group II.

**Table 3:** Comparison between groups.

	Group I	Group II	P Value	Effect Size (d)
UPDRS total score	-10.4±6.1	-4.2±4.5	< 0.01	1.16
UPDRS part II score	-2.8±1.9	-1.2±1.4	< 0.01	0.97
UPDRS part III score	-5.7±4.5	-1.9±3.3	< 0.01	0.98
TUG (s)	-4.9±5.6	-0.7±6.5	< 0.05	0.69
6MWT (m)	53.3±55.7	14.1±56.7	< 0.05	0.70
FOG score	-0.5±0.6	-0.1±0.3	< 0.05	0.91

Abbreviations: UPDRS, Unified Parkinson’s Disease Rating Scale; TUG, timed up and go test; BBS, Berg Balance Scale; 6MWT, 6-min walking; FOG, freezing of gait.

## Discussion

In the present study, we examined whether there was a difference in gait and balance ability between a group of PD patients treated with BWSOGT and a group treated with standard gait training to elucidate the effects of BWSOGT on motor abilities, such as gait and balance, in patients with PD. Group I showed significant improvements in total UPDRS, ADL, motor, TUG, and distance in the 6MWT relative to Group II. That is, this study showed that BWSOGT improved ADLs and motor ability, especially walking ability, walking endurance, and balance, more effectively than standard gait training for patients with PD.

BWSOGT improved the gait ability of patients with PD. BWSOGT supported the patient’s body weight and allowed the patient to walk with a stable trunk, which reduced the risk of falling and the patient’s risk of falling. Additionally, forward traction provided by an assistant increases patient stride length, which may improve the narrowed stride that occurs as a result of PD and improve gait. Increased stride length and faster gait for patients with PD improves their ability to walk [27–29]. BWSTT has been reported to increase stride length and improve gait in patients [8–10], and it is assumed that BWSOGT has a similar effect.

Nordic walking training has been reported to improve the 10-m and 6-min walks in patients with Parkinson’s disease [30, 31]. The H&Y scale values of the subjects in these studies are different from those in our study; therefore, their results cannot simply be compared with those of our study. Although the effect of Nordic walking on stride length has not been elucidated, we speculate that its application of poles expands the patient’s base of support, improves body stability, and increases stride length. Gait speed is strictly related to stride length. The low walking speed in patients with Parkinson’s disease is due to a reduction in stride length [32]. Both Nordic walking and BWSOGT increase self-selected walking speed, possibly via an increase in stride length.

BWSOGT allows for a rhythmic gait and increased hip extension angles and enables a faster gait [33]. It has been suggested that the spinal cord contains a central pattern generator (CPG) that provides motor neurons with cyclic motor output between flexor and extensor muscles, which is the basis of gait movements [34, 35]. This CPG is activated by rhythmic gait, hip extension movements, and faster gait [36]. Furthermore, compensatory excitation of the premotor and supplementary motor areas by CPG activity promotes functional reorganization of neural circuits [37–39]. It is assumed that BWSOGT promotes the activity of the motor output system on the basis of CPG and contributes to autonomous and coordinated gait reconstruction.

BWSOGT improved the TUG and 6MWT in patients with PD. It has been reported that training fast-moving repetitive movements in patients with PD improves their movement accuracy and enables them to make larger movements [40, 41]. It has also been reported that BWSTT increases patients’ stride length, gait speed, exercise tolerance, dynamic balance, and motor ability [16, 42–44], and that 20–30 minutes of moderate intensity exercise improves exercise tolerance [45]. These could be the mechanisms by which BWSOGT improved TUG and 6MWT in patients with PD.

BWSOGT improved UPDRS in patients with PD more than standard gait training. BWSTT for patients with PD improves motor ability and ADL performance. These are achieved because postural control is supported, which elicits freer movement of the lower limbs and alleviates the risk of falling [16, 46]. Rhythmic lower limb movements and sensory inputs to the lower limbs during gait training with body weight support promote motor leaning in patients with PD [37–39]. Furthermore, it has been reported that gait training in daily situations tends to be generalized to ADLs and practical gait [17]. It has also been reported that dynamic visual cues about the patient’s surroundings improve the effectiveness of gait training for motor learning and that audiovisual stimuli improve the effectiveness of gait training overall [11, 13]. These factors may have enhanced the training effect of BWSOGT and improved motor ability and ADL performance in patients with PD.

It has been reported that FOG is linked to gait asymmetry and coordination and postural instability [47–50] and is most likely to appear when turning and starting to walk [51]. Treadmill training with visual and auditory stimulation is effective in the treatment of FOG [8, 11], and it is assumed that training with external queuing and gait training with symmetrical and coordinated lower limb movements are effective. In our study, FOG was significantly improved in Group I, which may have contributed to the improvement in TUG and 6MWT performance. It has also been reported that treadmill walking enhances gait rhythm, reduces gait variation, and improves symmetry [37]. Furthermore, it has been reported that BWSOGT improves lower limb symmetry during gait more than treadmill walking [33, 52], which suggests the factor underlying the improvement of FOG in this study.

It has also been reported that short-term intensive training for 4 weeks improves clinical variables in patients with PD [12, 14] and that intensive effort-based repetitive training promotes neuroplasticity [53]. The training in this study was effective because it was a 4-week short-term intensive training program with effort-based repetitive training.

In this study, patients with H&Y stage IV were not excluded, suggests that BWSOGT may be effective even in patients with severe PD.

## Study limitations

Because the current study did not compare BWSOGT with treadmill walking, it was not possible to show whether BWSOGT or treadmill training was more effective. We also did not examine BWSOGT in terms of biomechanics.

Baseline UPDRS scores differed between groups. It is feasible that Group I individuals had greater disability than Group II; however, the difference was not statistically significant.

## Conclusions

BWSOGT for patients with PD improves gait ability and dynamic balance more than standard gait training.

## Supporting information

S1 Data (XLSX)

S1 Checklist STROBE statement—checklist of items that should be included in reports of case-control studies. (DOC)
